# The Interplay between Zinc, Vitamin D and, IL-17 in Patients with Chronic Hepatitis C Liver Disease

**DOI:** 10.1155/2015/846348

**Published:** 2015-10-04

**Authors:** Randa Reda, Amal A. Abbas, Mai Mohammed, Shahira F. El Fedawy, Hala Ghareeb, Rania H. El Kabarity, Rania A. Abo-Shady, Doaa Zakaria

**Affiliations:** ^1^Clinical Pathology Department, Faculty of Medicine, Ain Shams University, Abbassia Square, P.O. Box 11566, Cairo, Egypt; ^2^Tropical Medicine Department, Faculty of Medicine, Ain Shams University, Abbassia Square, P.O. Box 11566, Cairo, Egypt

## Abstract

*Objectives*. To assess zinc (Zn) and vitamin D (Vit. D) status in chronic Hepatitis C virus- (HCV) infected patients and their relationship to interleukin- (IL-) 17 and disease severity and then investigate whether Zn and Vit. D3 modulate IL-17 expression in chronic HCV patients. *Methods*. Seventy patients and fifty healthy subjects were investigated. Serum levels of Zn, Vit. D, and IL-17 were assessed in the patients group and subgroups. Patients lymphocytes were activated *in vitro* in the presence or absence of Zn or Vit. D3 and then intracellular IL-17 production was assessed using flow cytometry. *Results*. Zn and Vit. D were significantly decreased in HCV patients. Increasing disease severity leads to more reduction in Zn level opposed by increasing IL-17 level. Zn potently reduced IL-17 production in a dose-related fashion; however it did not exert any toxic effects. Although Vit. D apparently increases IL17 expression, it is unclear whether it is due to its toxic effect on cell count or lack of definite association between Vit. D and both IL-17 and disease severity. *Conclusions*. This study demonstrates that Zn modulates IL-17 expression and provides a rationale for evaluating this compound as a supplementary agent in the treatment of chronic HCV.

## 1. Introduction

Hepatitis C virus infects primarily the hepatocytes, leads to the development of fibrosis or cirrhosis of the liver, and is a significant risk factor for the development of hepatocellular carcinoma (HCC). Previous studies have demonstrated that T cell immunoregulatory cytokines contribute to liver damage [1].

Human interleukin-17 (IL-17) producing CD4 T cells, Th17, comprise a proinflammatory T cell subset. Previous studies have identified Th17 as a known arm of the CD4^+^ T-cell effector response [2] and several key cytokines, including IL-1, IL-6, tumor necrosis factor alpha, and IL-23, create a cytokine milieu that regulates the differentiation and expansion of human Th17 cell [3]. IL-17A can mobilize, recruit, and activate neutrophils, leading to massive tissue inflammation, and promote the progression of autoimmune disease. Furthermore, serum IL-17 levels are increased and serve as a marker of the severity of acute hepatic injury [4, 5].

Zinc, one of the essential trace elements, is required by many enzymes and transcription factors for their activity or the maintenance of their structure. It has a variety of effects in the immune system. Zn deficiency causes an imbalance between Th1 and Th2 function in periphery. Production of IFN-gamma and IL-2, Th1 products, is decreased, whereas production of IL-4, IL-6, and IL-10 Th2 products is not affected [6].

The main zinc metabolism occurs in the liver hepatocytes. In patients with Zn deficiency, reduced Zn concentrations in the liver are one of the causes of impaired hepatocytes regeneration [7]. It has been demonstrated that Zn may play an important role as a negative regulator of HCV replication in genome length RNA-replicating cells. Thus zinc supplementations appear to offer a novel approach for further strategies in treatment of intractable chronic hepatitis C [8]. Zinc uses are claimed to suppress Th17-mediated autoimmune diseases at least in part by inhibiting the development of Th17 cells via attenuating STAT3 activation [9].

Vitamin D is emerging as a critical factor involved in the regulation of the immune system, inflammatory response, and fibrogenesis [10]. Several studies on Egyptian patients with hepatitis C virus showed a significant reduction of Vit. D and its active metabolite in HCVg4-infected patients compared to healthy controls [11, 12]. Moreover they found a significant negative correlation between viral load and Vit. D status. Interestingly, low vitamin D levels have been related to poor liver function and stage of cirrhosis [10, 13]. Vitamin D was shown to reduce the expression of collagen and profibrotic factors leading to decreased fibrosis [14]. The effect of Vit. D on the behavior of Th17 cells has been investigated in different diseases and Vit. D suppresses the expression of IL-17 and IL-23 [12, 15, 16], the regulatory effect on Th17 cells by Vit. D occurs through the reduction of retinoic acid-related orphan receptor (ROR)*γt* expression [15]. Therefore, this study was designed to assess Zinc and Vit. D status in chronic HCV-infected patients and its relationship to levels of IL-17 as immune inflammatory mediators and to clarify the effect of Zn and Vit. D in modulating the expression of IL-17* in vitro*.

## 2. Materials and Methods

### 2.1. Subjects

Prior to initiation, this study received approval by the Ethical Committee of the Faculty of Medicine, Ain Shams University. The study recruited 70 patients with chronic HCV infection and they were diagnosed retrospectively by positivity of PCR and enzyme-linked immunosorbent assay (ELISA) HCV antibodies, who were selected from outpatients and inpatients of Internal Medicine and Tropical Medicine Departments at Ain Shams University Hospitals. In addition fifty healthy normal persons matched for age and sex as a control group were also included in the study. Inclusion criteria were based on a history of liver disease with HCV genotype 4 infection (as new patients or under follow-up). Patients with other causes of viral hepatitis: hepatitis B virus (HBV) or coinfection with HBV and human immunodeficiency virus (HIV), cytomegalovirus (CMV), and Epstein–Barr virus (EBV), or having criteria suggestive of fatty liver: Body Mass Index (BMI) >35, uncontrolled DM (HbA1c > 7), or history of taking hepatotoxic drugs for the previous 6 months, were excluded. BMI was calculated as weight in kilograms divided by the square of height in meters [17]. (Obesity was defined as BMI > 30 kg/m².)

All included patients underwent tests for liver function (ALT, AST, and bilirubin using Beckman Synchron CX7 Delta Clinical System, prothrombin time, and INR using stago analyzer), assessment of HCV levels, and abdominal ultrasonography for detection of cirrhosis. Based on ultrasonography results and liver function tests, the patients were classified into three subgroups:* Group 1* includes 16 recently diagnosed chronically HCV-infected patients showing no evidence of hepatic cirrhosis or liver cell failure.* Group 2* includes 37 compensated chronically HCV-infected patients showing an evidence of hepatic cirrhosis but no evidence of liver cell failure.* Group 3* includes 17 decompensated chronically HCV-infected patients showing an evidence of hepatic cirrhosis and liver cell failure.

### 2.2. Measurement of Serum Interleukin-17, Zinc Level, and 25-OH Vitamin D

After subclassification, venous blood samples (5 mL) were obtained (after overnight fasting) from all patients and controls. Samples were allowed to clot and sera were then separated by centrifugation (3500 rpm, 20 min, 25°C) and then stored at −20°C until used for serum analysis of the various parameters outlined below.

Commercially available ELISA kits (Labs Biotech, Inc, USA) were used for quantitative analysis of interleukin-17 while determination of zinc level was done by zinc colorimetric method (kit supplied from Química Clínica Aplicada S.A). Measurement of serum 25-OH vitamin D (as 25-OH vitamin D is the major circulating form of vitamin D and is used as an indicator of vitamin D status) using a commercially available (ELISA) kit supplied by Calbiotech's, Inc, USA, was done for 20 selected patients from Group 2. Vitamin D deficiency was defined as a 25(OH) D serum level < 12 ng/mL, vitamin D insufficiency as 25(OH) D level 12–32 ng/mL, and vitamin D sufficiency as > 32 ng/mL [18].

### 2.3. Assessment of Zn and Vit. D Effect on the Expression of IL-17 in Cultured PBMCs

#### 2.3.1. Reagents

Phorbol 12-myristate 13-acetate (PMA) and ionomycin (IO) were both purchased from Serva Electrophoresis Germany, zinc sulphate was purchased from Elnasr, Pharmaceutical Chemical Industries, Egypt, vitamin D3 (Cholecalciferol) from Memphis, Pharmaceutical Chemical Industries, Egypt, and Intracellular Fixation & Permeabilization Buffer (plus Brefeldin A) kit from eBioscience, San Diego, CA, USA.

#### 2.3.2. Monoclonal Antibodies

Antibodies used included CD3-PECy5, CD4-FITC, IL17-PE, and PE isotype control (eBioscience, San Diego, CA, USA).

#### 2.3.3. Preparation of Peripheral Blood Mononuclear Cells (PBMCs)

Twenty mL of peripheral blood were obtained by venipuncture from the 20 selected patients of Group 2 patients and collected into sterile EDTA tubes. The PBMCs were immediately separated by density gradient centrifugation over Ficoll–Hypaque (Lonza, BioWhittaker) and then washed twice with RPMI 1640. Cell count and viability were determined utilizing Guava ViaCount Flex Reagent for Flow Cytometry (Merck Millipore, France). Viability was exceeding 95% in all studied cases. PBMCs were suspended in RPMI 1640 medium, supplemented with 2 mM l-glutamine, 25 mM HEPES, 100 U/mL benzyl-penicillin, 0.1 mg/mL streptomycin, and 10% ABserum (complete medium) (Lonza, BioWhittaker). All cultures were incubated.

#### 2.3.4. Intracellular Cytokine Staining and Flow Cytometry

Peripheral blood mononuclear cells (PBMCs) from patients were cultured at a concentration of 5 × 10^5^/well in 200 *μ*L of complete medium in 96-well U bottom cell culture plates (Corning Incorporated, Corning, NY) and stimulated with 10 ng/mL of PMA plus 1 *μ*g/mL IO, in the presence or absence of vitamin D3 in two different concentrations (low 50 ng/mL and high 500 ng/mL) in some wells or Zn in the form of zinc sulphate in both low and high conc. (3 *μ*mol/L and 30 *μ*mol/L.) in other wells. The cells were incubated for 1 hour before the addition of Brefeldin A in a humidified incubator at 37°C and 5% CO_2_. Then, the incubation was continued for an additional 72 hours in the same circumstances. After incubation, the cells were washed twice with FACS buffer and stained for surface markers by incubation with CD3-PECy5 and CD4-FITC antibodies for 20 min in the dark at 4°C. Cells were then washed twice with FACS buffer and resuspended in fix buffer for 30 min at  4°C in dark followed by 2-time wash with diluted PERM buffer. The permeabilized cells then stained for intracellular cytokine (IL17) using IL-17-PE antibodies then incubated in the dark at room temperature for 30 min. After intracellular cytokine staining, the cells were washed and resuspended in 200 *μ*L phosphate-buffered saline. Flow cytometry was then performed and data was collected using a four-color Guava cytometer (Merck Millipore, France) and analysis was performed using FlowJo software (TreeStar, La Jolla, USA). The appropriate isotype-matched monoclonal antibodies were used to establish gating parameters.

### 2.4. Assessment of HCV Levels

Quantitative reverse transcription polymerase chain reaction (RT-PCR) for HCV was done using TaqMan technology by Stratagene Mx3000P Real-Time PCR System (Life Technologies, Applied Biosystems, USA); the RNA Isolation Kit (QIAamp minikit) and the reverse transcription and amplification Kit (Brilliant HCV QRT-PCR kit) were both purchased from Qiagen, Hilden, Germany. The RT-PCR had a limit of quantification (LOQ) of 25 IU/mL and a limit of detection (LOD) of 12 IU/mL.

### 2.5. Statistical Analyses

Data was analyzed using Prism 5 software (GraphPad, La Jolla, CA). Patient and control groups were compared using Student's* t*-test for parametric data while two-tailed Mann-Whitney and Kruskal-Wallis test were used for non parametric data. Correlations between parameters were determined using Spearman's correlation coefficient.

## 3. Results

### 3.1. Baseline Characteristics of Chronic HCV Patients

Seventy chronic HCV patients were enrolled. Demographical and clinical characteristics of patients and controls are reported in Table 1.

### 3.2. Elevated Levels of Serum IL-17 in Chronic HCV Patients Correlate with Severity of Liver Disease

**Figure 1:**
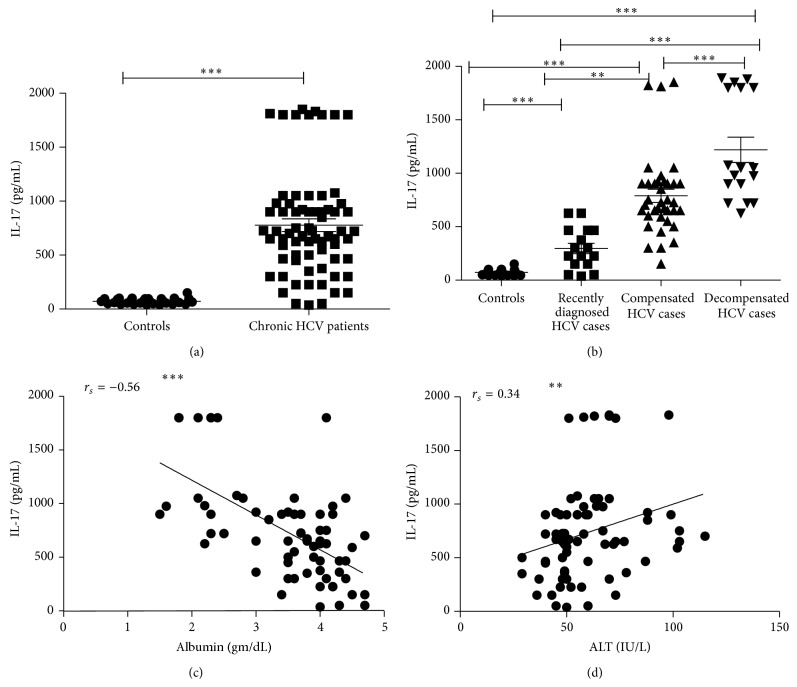
Serum IL-17 comparative results from chronic HCV patients and uninfected controls (a) and comparison between patients subgroups and controls (b). ∗ indicates *P* < 0.05; ∗∗ indicates *P* < 0.01; and ∗∗∗ indicates *P* < 0.001. IL-17 correlation with Albumin (c) and ALT (d). Spearman coefficients were determined (*r*_*s*_ = −0.56 for Albumin; and *r*_*s*_ = 0.34 for ALT).

Interleukin-17 is a potent mediator of delayed type reactions. It achieves this effect by elevating chemokine production in various tissues which, in turn, leads to recruitment of monocytes and neutrophils to the site of inflammation [19]. IL-17 was measured in the serum of chronic HCV patients (*n* = 70) and controls (*n* = 50). We observed significantly higher concentrations of IL-17 in patients group compared to control group (*P* < 0.001) (Figure 1(a)). To examine whether IL-17 was related to liver inflammation and fibrosis we stratified patients based on ultrasonography results and liver function tests. When comparing Strata, the recently diagnosed, compensated, and decompensated groups showed significantly higher levels of IL-17 as compared to controls; meanwhile the compensated and decompensated groups showed significantly higher levels of IL-17 when compared to recently diagnosed HCV group with *P* value < 0.001. Decompensated group also showed significantly higher levels of IL-17 as compared to compensated group (Figure 1(b)). In addition correlations between IL-17 and different laboratory parameters in patients group were done to show a significant negative correlation between IL-17 concentration and Albumin and a significant positive correlation with ALT, total and direct bilirubin, P.T, and INR (*P* < 0.001) (Table 2 and Figures 1(c) and 1(d)).

### 3.3. Zinc and Vitamin D Status in Chronic HCV Liver Disease and Their Relation with IL-17

**Figure 2:**
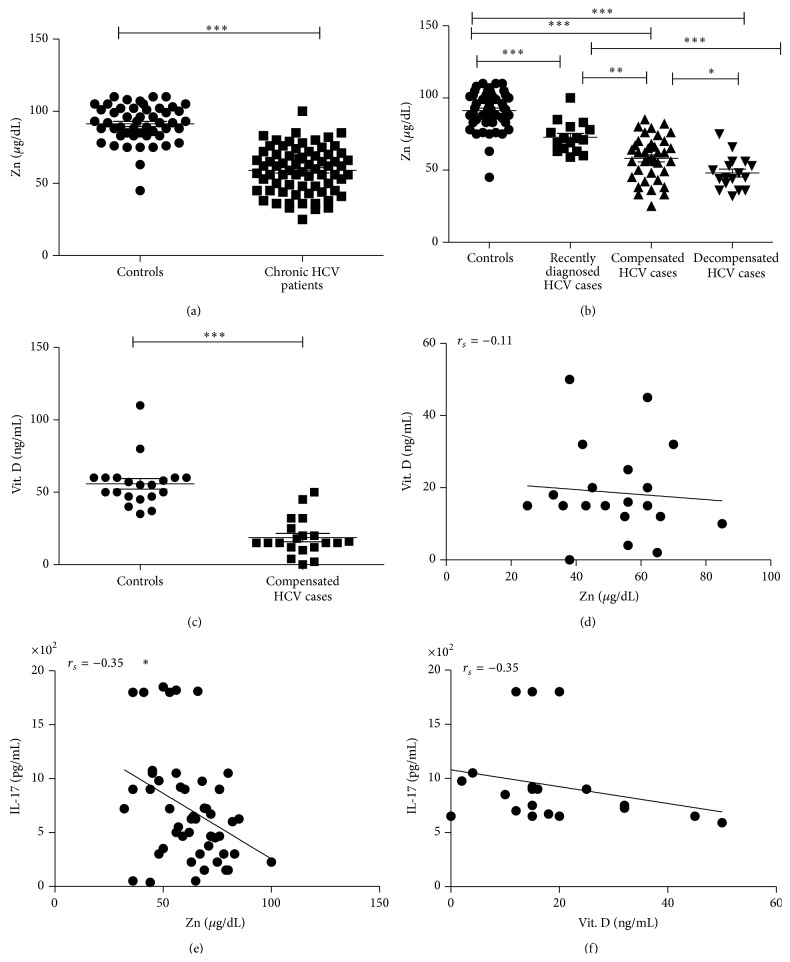
Lower Zn level in chronic HCV patients (a). Statistically significant differences in Zn level across the different groups are indicated (b). Lower Vit. D level in the selected compensated patients compared to controls (c). Correlation between Zn and Vit. D was nonsignificant Spearman coefficient (*r*_*s*_ = −0.11) (d). IL-17 was correlated with Zn (e) and Vit. D (f) and Spearman coefficient was calculated (*r*_*s*_ = −0.35). ∗ indicates *P* < 0.05; ∗∗ indicates *P* < 0.01; and ∗∗∗ indicates *P* < 0.001.

The serum levels of zinc are often decreased in HCV patients, and serum levels also tend to negatively correlate with hepatic reserve [20]. This was obviously noticed in the patients group and subgroups which showed highly significant decrease in Zn level when compared to control group (Figure 2(a)). In addition data shown in Figure 2(b) indicate that compensated and decompensated groups had lower Zn levels compared to recently diagnosed group. Decompensated group also showed significantly lower Zn levels as compared to compensated group. Moreover there was a significant positive correlation between Zn and Albumin and significant negative correlation with ALT, total and direct bilirubin, P.T, and INR (Table 2). Because the liver plays a central role in Vit. D metabolism and its inadequacy is common in chronic liver diseases and correlates with disease severity [21], we selected 20 chronic HCV cases from the compensated group to assess Vit. D status which was significantly lower than the controls level (Figure 2(c)); however no correlations were found between vitamin D serum levels, biochemical and virological data of the patients (data not shown), as well as serum Zn level (Figure 2(d)). We determined a significant negative correlation between serum level of Zn and IL-17 (Figure 2(e)). There is also negative correlation between serum Vit. D level and IL-17; however, it is not significant (Figure 2(f)).

### 3.4. Role of Both Zn and Vit. D in Controlling IL-17 Expression

**Figure 3:**
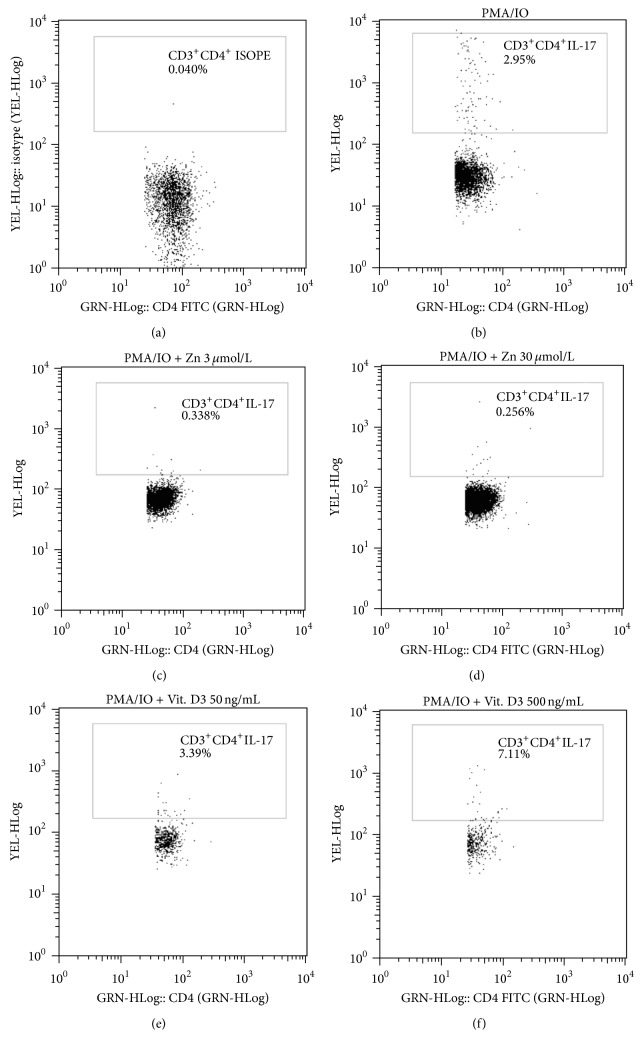
Representative flow cytometry plots for one patient are shown and gating of CD3^+^CD4^+^ ISOPE is indicated (a). Phorbol 12-myristate 13-acetate and ionomycin-stimulated peripheral blood mononuclear cells were stained and analyzed for IL-17 production (b). Inhibitory effect of Zn on IL-17 production with 3 *μ*mol/L concentration (c) and 30 *μ*mol/L concentration (d). Vitamin D3 effect on IL-17 production with 50 ng/mL concentration (e) and 500 ng/mL concentration (f).

**Figure 4:**
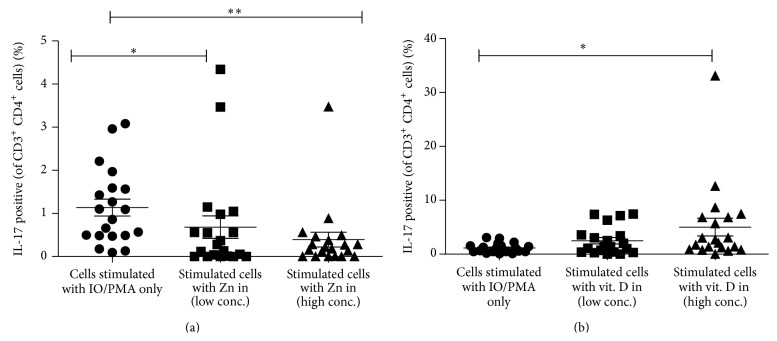
Peripheral blood mononuclear cells were stimulated with Phorbol 12-myristate 13-acetate and ionomycin in the presence or in the absence of Zn and Vit. D and stained and analyzed as described in the legend of Figure 3. Inhibition of IL-17 production by Zn in low (3 *μ*mol/L) and high (30 *μ*mol/L) concentrations (a). Significant increase in IL-17+ cells in the presence of high concentration (500 ng/mL) of vitamin D3 (b). ∗ indicates *P* < 0.05 and ∗∗ indicates *P* < 0.01.

Both vitamin D and zinc play a role in innate and adaptive immune responses controlling inflammatory cytokine gene expression [15, 22]. To test the effects of Zn and vitamin D3 on IL-17 cytokine production, PBMCs from only 20 compensated cases were stimulated with PMA plus IO, in the presence or absence of Zn (3 and 30 *μ*mol/L) or vitamin D3 (50 ng/mL and 500 ng/mL) then stained for intracellular IL-17, and analyzed by FACS (Figure 3). Data shown in Figure 4(a) indicate that the percentage of CD3^+^CD4^+^IL17^+^T lymphocytes was significantly lower in the presence of Zn than in its absence and became more lower with increasing Zn concentration which further support our suggestion regarding the link between Zn and IL-17. On the opposite side addition of Vit. D (in high concentration 500 ng/mL) leads to a significant increase in the percentage of CD3^+^CD4^+^IL17^+^ cells compared to its absence (Figure 4(b)).

### 3.5. Effect of Zn and Vitamin D3 on Lymphocyte Count

**Figure 5:**
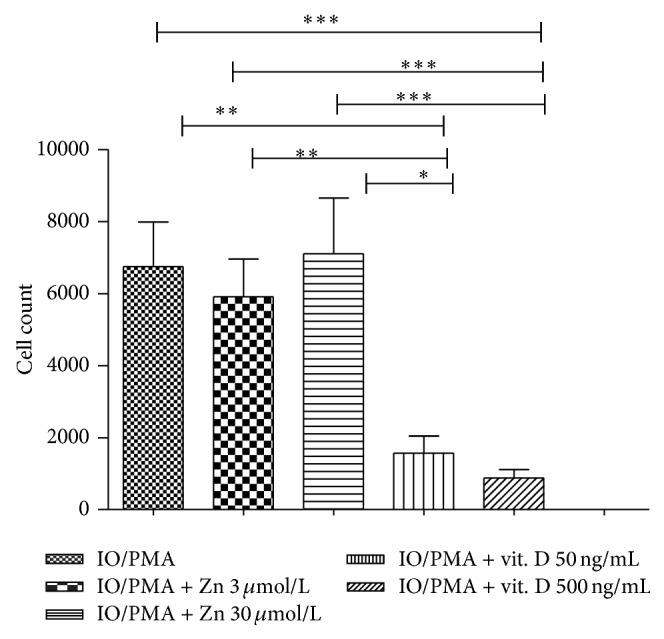
Different effect of Zn and Vit. D on cell count. Significant decrease in cell count in the presence of Vit. D in either concentration compared to its absence or compared to the presence of Zn.

In order to assess the* in vitro* effect of Zn and Vit. D on cell count we compared the count of cells which stimulated in the presence of Zn or Vit. D with those which stimulated in their absence. We noticed that addition of Zn leads to increase in the cell count especially with increasing the dose; however this effect was not significant; contrary to this result, addition of Vit. D leads to significant lowering in the cell count compared to cells stimulated in its absence or in the presence of Zn, and this effect seemed to be also dependent on the dose of vitamin D; however the *P* value was nearly significant = 0.057 (Figure 5).

## 4. Discussions

Hepatitis C virus infection is a significant global public health problem. Persistent HCV infection eventually develops into liver cirrhosis or hepatocellular carcinoma [23]. Many reports in HCV infections indicate a close correlation between virus-induced liver inflammations, infiltration, and activation of Th17 cells and the amount of liver damage caused by the antiviral immune response. Moreover a shift from Th1 to Th17 seems to be potentially disadvantageous for the patient in terms of antiviral defense and liver disease progression [24, 25]. The present study showed that IL-17 was markedly increased in HCV-infected patients in comparison to controls and this elevation became more evident with the progression of the disease as shown upon comparing patients' strata. These results were supported by the finding that increasing circulating Th17, intrahepatic IL-17 positive cells, and HCV-specific Th17 cells were correlated with severity of liver inflammation in chronic HCV patients [26]. IL-17 and Th17 seem to have an important role in viral infections and stronger Th17 responses are associated with higher viral plasma load, increased levels of serum transaminases, and enhanced activation of blood monocytes as well as liver macrophages [25]. Finally, it has been reported that antiviral therapy with pegylated interferon and ribavirin in HCV-infected patients leads to a reduction of both Th1 and Th17 responses, ameliorating HCV-mediated liver inflammation [27]. These studies explain the significant negative correlation between serum IL-17 and Albumin and the significant positive correlations with serum bilirubin, prothrombin time, ALT, and AST which were evident in the current study; however, we could not find any significant correlation between viral loads and serum IL-17 level. This finding was strengthened by Chang et al. [26] who found that no association was found between serum IL-17 concentration and viral load as viral loads fluctuate in chronic HCV infection. Taken together that many IL-17 sources might account for serum IL-17 levels, since IL-17 is produced by various cells (e.g., neutrophils, CD8^+^ T cells, *γδ* T cells, natural killer T (NKT) cells, and Tregs) [28], this in turn could account for the lack of correlation between serum IL-17 levels and viral load.

Zinc is a micronutrient influencing growth and affecting the development and integrity of the immune system. Furthermore, it plays an important role in the function of the liver [29]. Liver disease has been associated with hypozincemia and zinc deficiency [30]. Confirming prior observations we detected significant decrease in Zn level in the patients group compared to controls which was prominently noticed in decompensated disease stage compared to earlier stages. We explained these results on the basis that, in liver disease, there is increase in gut permeability with endotoxemia, infections such as spontaneous bacterial peritonitis, and release of stress hormones which could be the causes of Zn deficiency [31]. Moreover this stress response is often associated with hypoalbuminemia [32]. In consistency with this finding, we observed a positive correlation between serum Zn and Albumin levels as it is a major binding protein for Zn; however serum Zn concentration can decrease with an inflammatory stimulus even in the absence of hypoalbuminemia due to the effect of proinflammatory cytokines believed to play an important role in determining trace element concentration [33]. All in all from the previous observations we can understand the bases of the significant negative correlation between Zn and IL-17 which was obviously noticed in our result.

Regarding the role of Zn in HCV infection it may act as a negative regulator of HCV replication in genome-length HCV RNA-replicating cells; thus its deficiency may increase the severity of the case [8]. In addition it was found that Zn levels depend on the severity of liver disease; thus, in patients with decompensated liver cirrhosis, Zn concentrations are reduced by up to 75%, this is explained by changes in the protein and amino acid metabolism and by disturbances in intestinal resorption and hepatic Zn extraction [33]. These data explain the highly significant negative correlation we found between zinc level and the other liver function tests; however we could not mount a significant correlation between viral loads and serum Zn level.

Vitamin D is a potent immunomodulator that favors innate immunity and cell differentiation [34, 35]. Increased production of 1,25-dihydroxy vitamin D3 results in the synthesis of cathelicidin, a peptide capable of destroying many viral infectious agents. Low serum levels of vitamin D prevent macrophages from initiating innate immune response [36]. Vitamin D deficiency is very common (92%) among patients with chronic liver disease, and at least one-third of them suffer from severe vitamin D deficiency [37]. Moreover levels of Vit. D and its active form are significantly lower in advanced liver disease (hepatic cirrhosis and/or carcinoma) patients, compared to those with bright hepatomegaly and perihepatic fibrosis [12]. In the light of these findings we assessed 25-OH Vit. D level in twenty compensated HCV patients which showed a significant reduction compared to healthy controls. This decrease in Vit. D was concomitant with an increase in IL-17 levels (however the negative correlation between them was not significant); this could be attributed to the finding that Vit. D suppresses proinflammatory cytokines and increases anti-inflammatory cytokines [38]. Vit. D regulates the growth and differentiation of multiple cell types and displays immunoregulatory and anti-inflammatory properties. Cells involved in innate and adaptive immune responses including macrophages, dendritic cells, T cells, and B cells express vitamin D receptor (VDR) and can both produce and respond to 1,25(OH)_2_D_3_ [39]. Hepatocytes express only low levels of VDR mRNA [40], so that vitamin D effects on the liver are most probably not conferred by direct signalling in parenchymal liver cells. In contrast, nonparenchymal hepatic cells such as sinusoidal endothelial cells, Kupffer cells, and hepatic stellate cells (HSC) do express VDR mRNA and functionally active VDR protein [41]. Many reports indicate that 1,25(OH)_2_D_3_ suppresses Th17 driven cytokine responses, induces Treg cells, induces IL-4 production (Th2), and enhances natural killer T-cell function [11, 42] but the key immunomodulatory property of 1,25(OH)_2_D_3_ is its ability to inhibit expression of Th1 cytokines, whilst augmenting Th2 cytokines, with 1,25(OH)_2_D_3_ acting either directly via effects on T lymphocytes or indirectly via effects on antigen-presenting cells (APCs). Moreover, elevated VDR expression is also found on differentiated Th17 cells [43]. More recent studies showed marked anti-inflammatory and antifibrotic effects of VDR-signalling in HSC. During inflammatory liver injury after endotoxin injection, the activation of VDR signalling by vitamin D attenuated liver damage* in vivo*. Vit. D provides protection against autoimmune and inflammatory diseases, such as multiple sclerosis, type 1 diabetes, and inflammatory bowel disease partially due to its inhibitory effects on Th17 cells. The antiproliferative, prodifferentiative, antibacterial, immunomodulatory, and anti-inflammatory properties of synthetic VDR agonists could be exploited to treat a variety of inflammatory and autoimmune diseases [39]. Treatment with VDR agonists inhibits the T-cell production of IL-17. Furthermore, IL-17 production is sustained by IL-23, an IL-12 family member, the latter of which is strongly inhibited by VDR agonists [11]. It was clearly demonstrated that enhanced progression of liver fibrosis is significantly and independently associated with both genetic VDR variants and low 25-OH vitamin D plasma levels. This suggests vitamin D substitution as a preventive measure for patients with liver fibrosis [44].

In order to gain additional insight into the interplay between Zn, Vit. D, and IL-17 we have examined the ability of Zn and Vit. D3 to interfere with Th17 activation and expression of IL-17* in vitro*. Noteworthy, IL-17 was suppressed significantly when Zn was added in both concentrations and this was claimed to its suppressive effect on IL-6/STAT3 (signal transducer and activator of transcription) signaling pathway which is a critical step for Th17 development. Zn binding changed the* a*-helical secondary structure of STAT3, disrupting the association of STAT3 with JAK2 kinase (Janus kinase 2) and with a phosphopeptide that included a STAT3-binding motif from the IL-6 signal transducer gp130 [22]. To test whether the effects of Zn on cytokine production were independent of toxic effects, cell count was examined and interestingly we observed that, with addition of Zn, the count was increased compared to its absence especially with increasing Zn concentration, although this observation was not significant; this effect could be mediated through Zn enhancement of DNA synthesis and RNA transcription, cell division, and cell activation as apoptosis (programmed cell death) is potentiated by Zn deficiency [45]; additionally Zn is an inhibitor of NADPH oxidases which catalyze the production of reactive oxygen species (ROS); on the other side, Zn activates the dismutation of O2^·−^ to H_2_O_2_ by super oxide dismutase which contains both copper and Zn [46]; it also negatively regulates gene expression of inflammatory cytokines such as TNF-*α* and IL-1*β*, which are known to generate (ROS) and this may be one additional mechanism by which Zn may be functioning as an antioxidant in humans [47].

There is promising evidence that zinc may decrease liver injury and provides antifibrotic effects in patients with chronic HCV. Himoto and Coworkers [20] used polaprezinc as an antifibrotic therapy in patients with chronic HCV and showed a decrease in noninvasive fibrosis markers.

One caveat of our studies was the effect of Vit. D on IL17 production. For reasons that remain unclear and despite the apparent negative association between serum Vit. D and IL17, addition of Vit. D showed an apparent increase in the percent of IL-17^+^ cells which was significant only with high concentration of Vit. D. Contrary to our finding an experimental study on healthy human donor using CD4^+^ T cells and mouse model for multiple sclerosis showed that 1,25(OH)_2_D_3_ inhibits human IL-17A and suppresses mouse IL-17A [48]; other studies in mice imply this regulatory effect on Th17 cells by Vit. D through the reduction of (ROR)*γt* expression [15].

The reasons of these conflicting results still need to be determined, although we would contend that the interpretation was somewhat misleading. Looking at the effect of Vit. D on cell count showed us a marked dose-related reduction in cell count compared to its absence or to the presence of Zn. Preclinical research indicates that vitamin D3 potently inhibited T cell proliferation in a dose-related fashion [49]. The active metabolite of vitamin D, 1 alpha,25(OH)_2_D_3_, also known as calcitriol, has antiproliferative effects, activates apoptotic pathways, and inhibits angiogenesis [50]. The common antiproliferative vitamin D receptor (VDR) functions are associated with arrest at G0/G1 of the cell cycle, coupled with upregulation of a number of cell cycle inhibitors including p21 and p27 [51]. On the other hand Th17 and other Th17 and other IL-17 secreting cells could play a part in hepatic viral persistence bymeans of antiapoptoticmolecules upregulation [26]. Consistently, IL-17 has been implicated in modulating the expression levels of prosurvival Bcl-2 family proteins, including Bcl-2 (B-cell lymphoma 2) [52] and Bfl-1/A1 (Bcl-2 family member) in some autoimmune diseases such as systemic lupus erythematosus [53]. Based on these observations it seems feasible that our preconceived notion regarding the impairment of apoptosis pathway in Th17 cells can contribute to the rise of IL-17^+^ cells percent among the remained cells; however it remains to be determined whether the apparent increase is a true enrichment of IL-17 producing cells or simply due to survival of IL-17^+^ cells from the toxic effect of Vit. D. However the clinical implication of low vitamin D levels and HCV severity is still not clear. At least one study has found that serum Vit. D levels were not associated with any parameter indicating disease severity. And they concluded that serum Zn but not serum vitamin D levels is strongly associated with disease severity and treatment response in chronic HCV [54]. This latter study may also account in part for the lack of significant correlations between Vit. D serum levels, biochemical and virological data of the patients, and serum Zn and IL17 levels that have been investigated in the current study. However there are some limitations in this study. An important limitation is the lack of assessment of Vit. D status in both recently diagnosed and decompensated groups which would give us a better informative insight on the interplay between Vit. D and IL-17 in chronic HCV liver disease and whether it has a protective role in preventing liver fibrosis or not.

A final conclusion of our study concerns the potential use of Zn as an adjunct antifibrotic therapy and novel strategies for the treatment of chronic hepatitis C; it may be worthwhile exploring the benefit of zinc supplementation even with the advent of novel direct antiviral agents. Meanwhile the role of vitamin D is still a topic of debate and much work will be required to understand the perturbations found in IL-17 and its relation to vitamin D at a mechanistic and genetic levels.

## Figures and Tables

**Table 1:** Demographical and clinical characteristic of patients and controls.

Clinical characteristics	All patients	Recently diagnosed	Compensated	Decompensated	Controls
Age, median (range)	48 (22–76)	30 (22–35)	48 (40–54)	63 (53–76)	31 (27–40)
Sex (M/F)	35/35	9/7	17/20	9/8	22/28
Albumin (g/dL), median (IQR)	3.6 (2.4–4)	4.2 (4–4.4)	3.6 (3.5–3.8)	2.3 (1.95–2.45)	>4.1
Total Bilirubin (mg/dL), median (IQR)	0.9 (0.7–1.7)	0.7 (0.5–0.8)	0.7 (0.6–0.9)	3.14 (1.7–5.1)	<1.2
Direct Bilirubin (mg/dL), median (IQR)	0.3 (0.2–0.9)	0.2 (0.1–0.3)	0.2 (0.1–0.3)	1.07 (0.8–2.35)	<0.2
P.T# (sec), median (IQR)	13 (11.8–16.6)	11.9 (11.63–12.48)	12.5 (11.8–13)	17.4 (16.3–19.9)	<12
INR, median (IQR)	1.015 (0.98–1.5)	0.99 (0.9–1.04)	1 (0.9–1.07)	1.5 (1.48–1.7)	0.93 (0.9–1.02)
ALT (IU/L), Mean ± SD	53.84 ± 12.36	56.5 ± 13	47.59 ± 11.87	58 ± 10.22	15.5 ± 7.65
AST (IU/L), Mean ± SD	42.74 ± 10.59	47.44 ± 8.78	38.35 ± 9.32	42.71 ± 11.93	15.5 ± 6.25
PCR (IU/mL), median (IQR)	31850 (22100–599000)	27600 (22100–646000)	36100 (22100–599000)	27600 (21200–630000)	—
IL-17 (pg/mL), Mean ± SD	776.3 ± 57.39	295.8 ± 48.04	766.1 ± 62.90	1207 ± 113.8	72.17 ± 4.622
ZN (*μ*g/dL), Mean ± SD	59.01 ± 1.86	72.81 ± 2.679	58.13 ± 2.445	48.00 ± 2.727	91.28 ± 1.838
Vit. D (ng/mL), Mean ± SD^a^	—	—	18.65 ± 12.89	—	55.8 ± 16.27

^a^Data available for 20 patients.

P.T: prothrombin time; INR: international normalized ratio; ALT: alanine aminotransferase; AST: aspartate aminotransferase; IQR: the interquartile range; HCV: hepatitis C virus.

**Table 2:** Correlations between IL17 and Zn and different parameters in hepatitis C virus infected subjects.

	IL-17		Zn	
	*R*	*P* value	*R*	*P* value
Albumin (gm/dL)	−0.561	<0.001	0.616	<0.001
Total Bilirubin (mg/dL)	0.792	<0.001	−0.678	<0.001
Direct Bilirubin (mg/dL)	0.649	<0.001	−0.584	<0.001
P.T (sec)	0.656	<0.001	−0.632	<0.001
INR	0.484	<0.001	−0.557	<0.001
ALT (IU/L)	0.34	0.004	−0.304	0.032
AST (IU/L)	−0.059	0.684	−0.042	0.773
PCR (IU/mL)	−0.018	0.903	0.054	0.711
